# Solar cell design using graphene-based hollow nano-pillars

**DOI:** 10.1038/s41598-021-95684-2

**Published:** 2021-08-09

**Authors:** Shiva Hayati Raad, Zahra Atlasbaf

**Affiliations:** Department of Electrical and Computer Engineering, Tarbiat Modares University, Tehran, Iran

**Keywords:** Solar cells, Devices for energy harvesting

## Abstract

In this paper, the full solar spectrum coverage with an absorption efficiency above 96% is attained by shell-shaped graphene-based hollow nano-pillars on top of the refractory metal substrate. The material choice guarantees the high thermal stability of the device along with its robustness against harsh environmental conditions. To design the structure, constitutive parameters of graphene material in the desired frequency range are investigated and its absorption capability is illustrated by calculating the attenuation constant of the electromagnetic wave. It is observed that broadband absorption is a consequence of wideband retrieved surface impedance matching with the free-space intrinsic impedance due to the tapered geometry. Moreover, the azimuthal and longitudinal cavity resonances with different orders are exhibited for a better understanding of the underlying wideband absorption mechanism. Importantly, the device can tolerate the oblique incidence in a wide span around 65°, regardless of the polarization. The proposed structure can be realized by large-area fabrication techniques.

## Introduction

Solar energy trapping, as the main source of renewable energy, has been the focus of extensive research for many years^1^. For this purpose, different geometrical and material combinations have been proposed, only some of them covering the whole spectrum. For instance, the relatively poor absorption efficiency of thin-film solar cells is the consequence of the indirect bandgap of silicon, where surface plasmon resonances of metallic nanoparticles can be used to enhance it^2,3^. In these solar cells, prolonged action of air and moisture affect the photocurrent generation due to the formation of oxide layers on metallic particles^4^. As an alternative approach, the front-sided integration of high-index dielectric particles has been proposed to enhance the power conversion efficiency of the thin-film solar cells^5^. Metasurface-based absorbers are another common category of solar cells, where metal–insulator–-metal (MIM) stacks have been widely used. In the aforementioned solar thermo-photovoltaic systems (STPV), the proper choice of the metal (rather than using noble metals) to withstand high temperature is a key factor^6^. Refractory metals, possibly coated with protection layers, are good candidates for this purpose because of their heat-tolerant and large absorption capabilities^7–9^.

Because of their broadband absorption nature, excellent chemical stability, high thermal stability, and excellent thermal conductivity, carbon-based materials have gained lots of interest as photo-thermal materials^10,11^. In this regard, a graphene\semiconductor heterostructure has been proposed for stable and efficient conversion of the light to electricity, being much thinner than its commercial silicon p–n junction counterparts^12^. Moreover, silicon photovoltaics can benefit from graphene as a semi-transparent electrode and an antireflection coating^13^. As another instance, by integrating Al nanoparticles and wrinkle-like graphene sheets, 7.2% enhancement in the photocurrent density of the solar cell is attained^14^. Moreover, broadband absorption of the un-polarized light is achieved using a 90-nm graphene dielectric stack which behaves as a hyperbolic metamaterial^15^. The aim of the present research is the design of a graphene-based ultra-broadband absorber covering the whole solar spectrum. A comprehensive review of graphene synthesis and functionalization for solar cells can be found in^16^.

Ultra-broadband absorbers can be realized using different approaches. Combined narrowband resonances to form super-unit, multi-resonance structures, and multi-layered configurations are beneficial for resonant devices^17^. In the non-resonant approach, the wideband absorption is the consequence of gradient impedance matching, and the energy is dissipated either in lossy conductors or lossy dielectrics^18^. For instance, in the Sierpinski carpet, MIM absorber, and metallic nano-wires filled pyramid dielectric medium absorber, the absorption spectrum is widened by merging multiple resonances^19,20^. The absorption enhancement mechanism of the wideband truncated-pyramid-based metamaterial absorber and truncated titanium/semiconductor cone absorber can be respectively understood by extracting the effective impedance and Fabry–Perot cavity like resonances of the high-index dielectric resonator^21,22^. Due to the inherent wideband nature of tapered structures, in the paper, the cone-shaped geometry is chosen as the building block and its absorption spectrum is further manipulated by truncating the tip and hollowing the nano-pillars. In the following paragraphs, after wisely choosing the materials, the optical performance of the shell-shaped truncated hollow cone solar cell will be discussed in detail. The paper is organized as follows. At first, the potential of graphene material in the solar cell design is investigated by introducing its constitutive parameters. Later, the attenuation constant of the electromagnetic wave illuminated to the graphene slab is calculated to confirm its absorption capability. Finally, using a shell-shaped graphene-based nano-pillar and refractory metal substrate, respectively the impedance matching and transmission blockage conditions are satisfied in the desired spectrum. Multiple parametric studies are conducted to better understand the performance.

## Results and discussions

In the following sections, the design procedure and performance analysis of a graphene-based solar cell is investigated in detail. After exhibiting the absorption capability of graphene material in the solar spectrum, by wideband impedance matching and transmission elimination, a full-spectrum solar cell is proposed and its performance is discussed employing multiple simulations.

### Material choice

**Figure 1:**
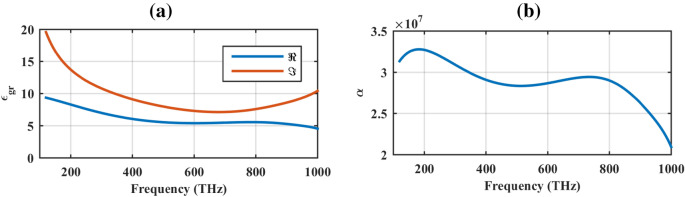
(**a**) Real and imaginary parts of graphene equivalent permittivity in the solar spectrum and (**b**) its attenuation constant.

Let us initially investigate the equivalent permittivity of graphene material in the solar spectrum. The solar radiation covers the wavelength range of 295–2500 nm (~ 100–1200 THz), comprised of ultraviolet, visible, and infrared light^9^. In the infrared regime, graphene is commonly modeled by its surface conductivity calculated based on Kubo formulas. The outstanding feature of graphene in the plasmonic state is being reconfigurable through its chemical potential and relaxation time^23^. In this band, graphene surface conductivity can also be converted to the equivalent bulk permittivity with the negative real part using the Ampere’s low^24^. In the visible spectrum and beyond, graphene behaves as an ordinary dielectric. Thus, the Drude Lorentz model can be used to approximate the monolayer graphene dispersive permittivity using the measured data^25^. Hence^26^:

1 $$ \varepsilon_{gr} = \varepsilon_{\infty } - \frac{{\omega_{gra}^{2} }}{{\omega^{2} + i\omega \gamma_{gra} }} + \sum\limits_{j = 1}^{m} {\frac{{\Delta \varepsilon_{j} \Omega_{j}^{2} }}{{\Omega_{j}^{2} - \omega^{2} - i\omega \Gamma_{j} }}} $$

where $m = 3$, $\varepsilon_{\infty } = 1.964$, $\Delta \varepsilon_{j} = \left( {6.99,\,\,1.69,\,\,1.53} \right)$, $\hbar \Gamma_{j} = \left( {7.99,\,2.01,\,0.88} \right)\,eV$, $\hbar \omega_{gra} = 6.02\,eV$, $\hbar \gamma_{gra} = 4.52\,eV$, $\hbar \Omega_{j} = \left( {3.14,\,4.03,\,4.59} \right)\,eV$. Figure 1a shows the real and imaginary parts of graphene permittivity in the solar spectrum, where relatively high real and imaginary parts are observed. In the next step, the attenuation constant of the illuminating wave to a graphene slab is studied to investigate whether the electromagnetic wave can penetrate it or not. The attenuation constant (Neper/meter) reads as^27^:

2 $$ \alpha = \omega \sqrt {\varepsilon_{0} \mu_{0} } \left( {a^{2} + b^{2} } \right)^{{{1 \mathord{\left/ {\vphantom {1 4}} \right. \kern-\nulldelimiterspace} 4}}} \sin \left( {\frac{1}{2}\tan^{ - 1} \left( \frac{a}{b} \right)} \right) $$

The parameters *a* and *b* in (2) are defined as: $a = \left( {\varepsilon^{\prime}_{r} \mu^{\prime}_{r} - \varepsilon^{\prime\prime}_{r} \mu^{\prime\prime}_{r} } \right)$ and $b = \left( {\varepsilon^{\prime}_{r} \mu^{\prime\prime}_{r} + \varepsilon^{\prime\prime}_{r} \mu^{\prime}_{r} } \right)$. As Fig. 1b confirms, the graphene material has a large attenuation constant in the solar spectrum, making it suitable for the absorber design. Where prime and double prime respectively denote the real and imaginary parts of the permittivity (*ε*) and permeability (μ). Graphene is a non-magnetic material, therefore the real and imaginary parts of its permeability are respectively one and zero.

### Proposing the device and performance analysis

**Figure 2:**
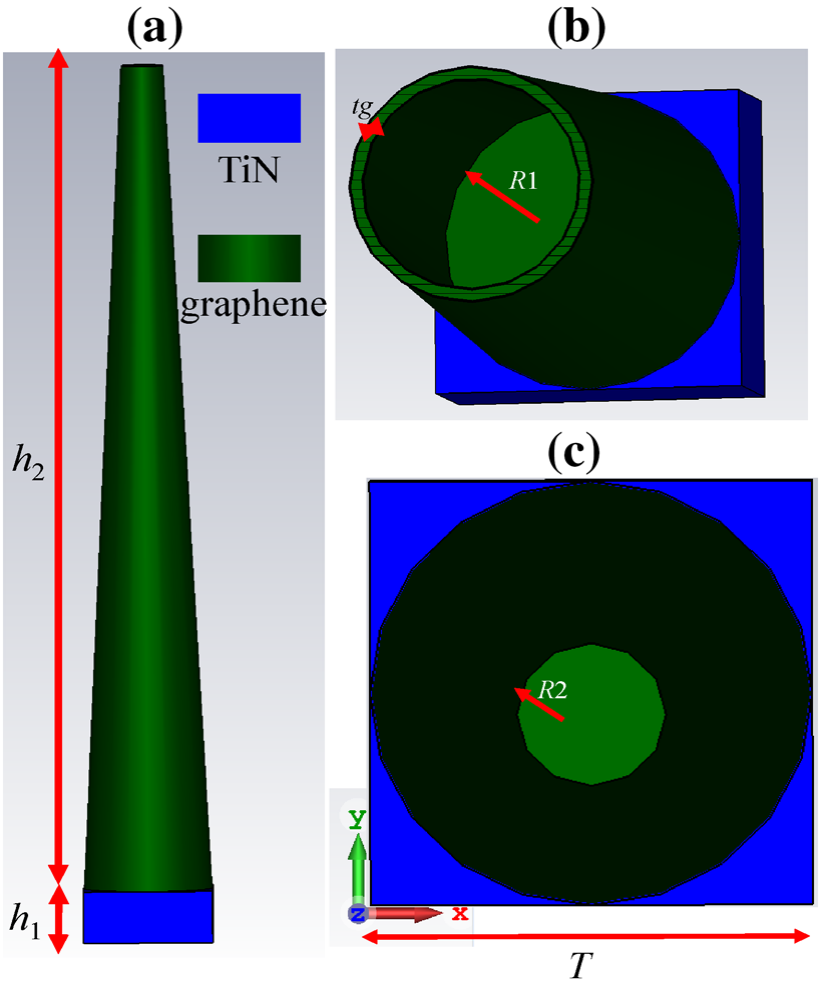
Unit cell of the proposed solar cell constructed by hollow graphene-based shell-shaped nano-pillars backed by a refractory metal (**a**) side view (**b**) top view for *h*_2_ = 500 nm and (**c**) overall top view. The substrate and nano-pillar heights are respectively *h*_1_ and *h*_2_. The thickness of the graphene shell is denoted by *t*_*g*_. Moreover, the bottom and top radii of the cone are *R*_1_ and *R*_2_ respectively. The periodicity of the nano-pillars in the square lattice is *T*.

The unit cell of our proposed absorber for the full coverage of the solar spectrum is illustrated in Fig. 2. This geometry is constructed by a refectory metal substrate, where its surface is covered by high aspect ratio graphene-based shell-shaped hollow nano-pillars. The height of the substrate and nano-pillars are considered *h*_1_ = 100 nm and *h*_2_ = 1600 nm, respectively. The dispersive permittivity of TiN refractory metal is extracted from^28,29^ and that of the graphene is based on (1). The graphene nano-pillar is with a bottom radius of *R*_1_ = 120 nm and a top radius of *R*_2_ = 40 nm. Moreover, the periodicity is *T* = 2*R*_1_ + *p* = 240 nm (*p* = 0) and the thickness of the graphene shell is *t*_*g*_ = 10 nm. The dispersive permittivity introduced in (1) is used for the layered graphene as well^30^. Note that 3D nano-pillars can be fabricated by hole-mask colloidal lithography^31^. The method can also be used for the large-scale fabrication of shaped high index dielectric patterns^32^. Tape-assist transfer and spin coating can be used for wrapping graphene on curved surfaces^33–35^. Moreover, the core template can be removed by immersing it in hydrofluoric acid (HF) solution with stirring to reach ultrathin-shell graphene hollow particle^36^.

**Figure 3:**
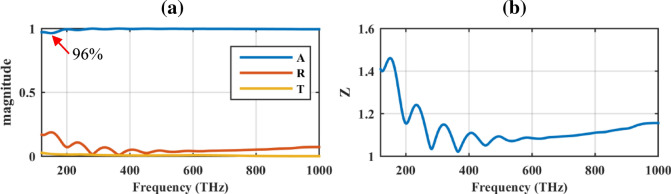
(**a**) The absorbance (*A*), reflectance (*R*), and transmittance (*T*) of the designed solar cell and (**b**) its retrieved surface impedance.

As Fig. 3a shows, the achieved absorption rate is above 96% in the whole solar spectrum, being above 99% beyond 247  THz. Included in the figure are the reflectance and transmittance curves, showing a negligible amount of them. Moreover, the retrieved surface impedance is exhibited in Fig. 3b. The results are provided based on the simulated scattering parameters via^37^:

3 $$ Z = \pm \frac{{1 + S_{11} }}{{1 - S_{11} }} $$

Based on the figure, wideband impedance matching is observed in this spectrum. The results of the above figures show that the underlying mechanism of the absorption is wideband impedance matching and transmission blockage^38^. The geometrical parameters are comparable with the TiN-shell based nano-pillar solar cell^31^.

The spatial components of the electric field at three different frequencies (begin, middle, and end of the spectrum) are provided in Table 1. As the table indicates, azimuthal and longitudinal cavity resonances with different orders are responsible for the high absorption rate. The field distribution at the lower frequency edge shows that the height of the cone can be tuned based on the minimum frequency requirement. Note that although high aspect ratio micro-nano structures are realizable^39^, the impact of the height on optical absorption is further discussed in the last section to reach geometrically compact devices for high-frequency operation.

**Table 1:**
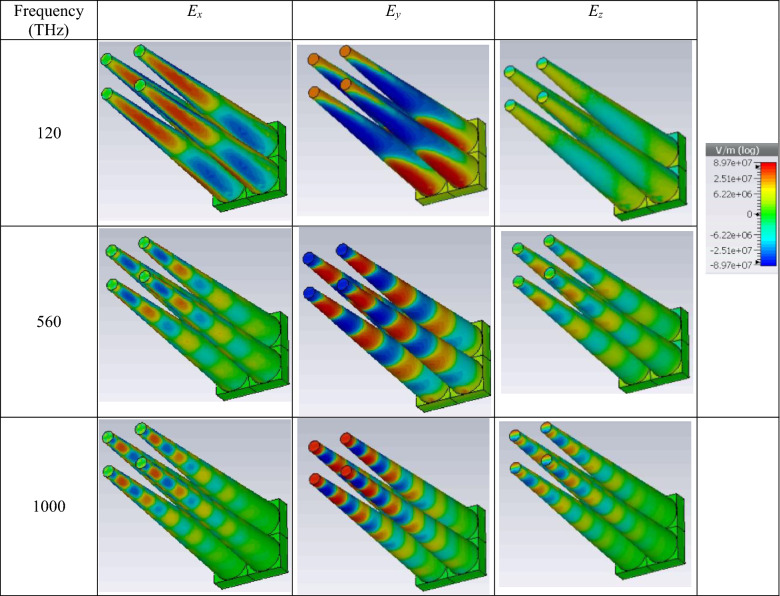
Spatial distribution of the electric field at three different frequencies.

**Figure 4:**
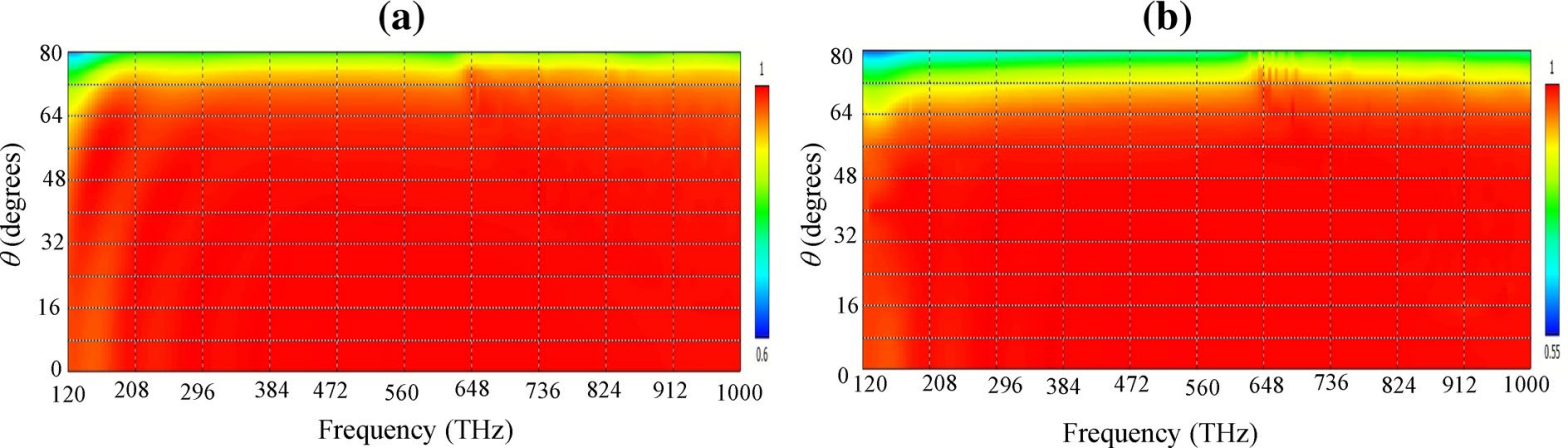
The sensitivity of the absorption spectrum to the incident angle for (**a**) TE and (**b**) TM waves.

The sensitivity of the absorption to the incident angle of the radiating wave is shown in Fig. 4 for TE and TM waves. The device has a polarization-insensitive response due to its four-fold symmetry^40^ and the absorption rate above 90% can be attained for angles up to 65°.

**Figure 5:**
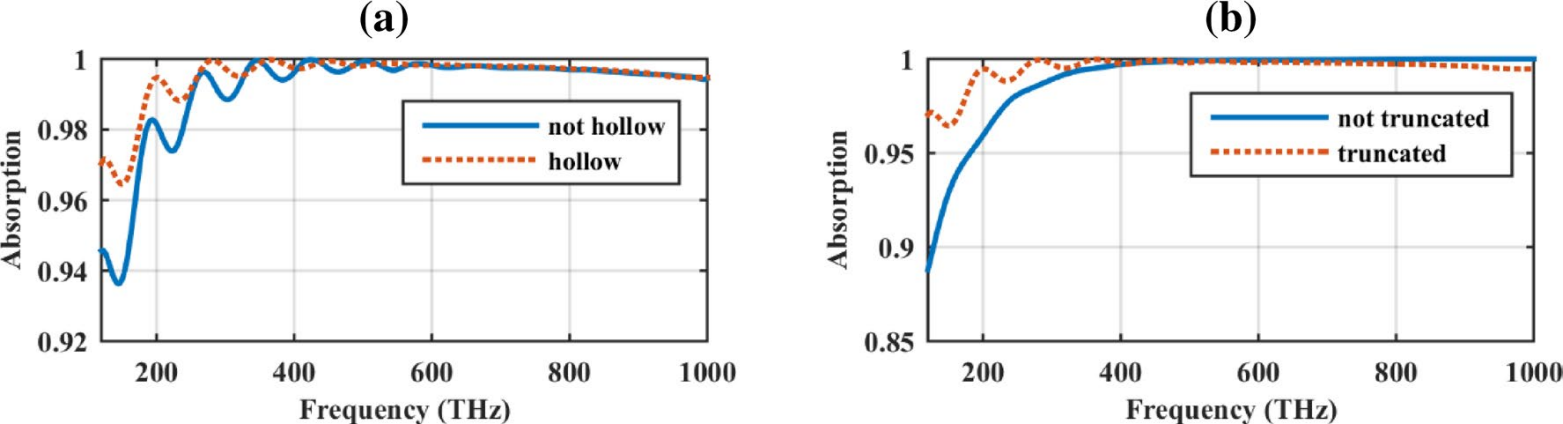
The absorption rate of the proposed solar cell when (**a**) filling the core with SiO_2_ and (**b**) not truncating the cone tip.

Figure 5a shows the absorption rate of the proposed solar cell when the core is made of SiO_2_ material with a refractive index of 1.45^9^. Based on the figure, the hollow nano-pillars have better low-frequency performance. Absorption rate enhancement by decreasing the core permittivity is expected since it is equivalent to the decrease in the size of the system^41^. The reader is referred to the experimental realization of carbon-based hollow particles for high power energy applications as further examples^42–45^ Moreover, in Fig. 5b, the impact of tip truncation in the low-frequency absorption improvement is observed.

### Parametric analysis

**Figure 6:**
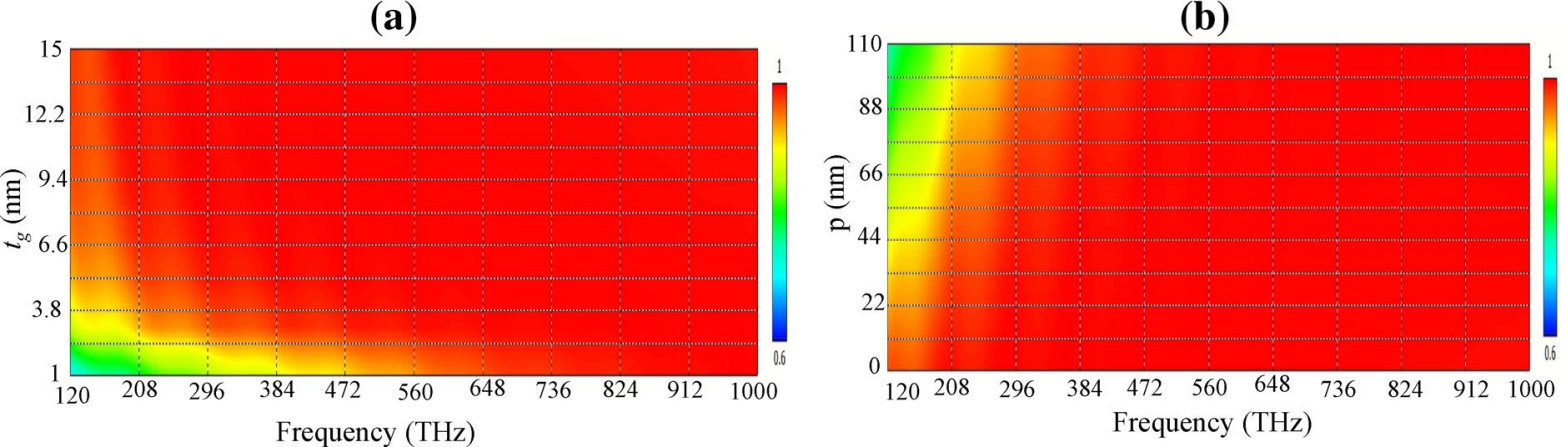
The absorption rate of the proposed solar cell for (**a**) different thicknesses of graphene shell *t*_*g*_ and (**b**) the periodicity of the nano-pillars. The parameter *p* is the increment in the periodicity from its minimum value 2*R*_1_ (*T* = 2R_1_ + *p*).

The optical absorption of the proposed device with changes in different geometrical parameters is studied in this section. Initially, let us consider the impact of the thickness of the graphene shell on the absorption rate. The reported absorption manipulation by the increase in the number of wrapped graphene layers around a spherical resonator exhibit different performance for solid silver and dielectric-metal core–shell resonators, respectively increasing and decreasing for thicker shells^30^. As Fig. 6a shows, the mono/few-layer graphene material has a low absorption rate at low frequencies. Thus, the presence of around 10 graphene layers, each with a thickness of 1 nm, is essential for the full spectrum coverage in the proposed device. Note that graphene is essentially a 0.34 nm monolayer of carbon atoms, but inhomogeneities during the fabrication process may result in thicker graphene sheets. For CVD-grown graphene material, treating the monolayer graphene as 1 nm thick effective material is a reasonable choice^46^. Using few-layer graphene films in the optical design, cost, scalability, and ultra-broadband absorption features can be attained but the effective transport of the photothermal energy generated on the surface and the possibility of performance manipulation become limited^11^. Moreover, Fig. 6b investigates the influence of inter-particle distance in the performance and shows that near-field coupling plays a crucial role in the absorption enhancement at lower frequencies since by increasing the distance between the nano-pillars the low-frequency performance is highly affected.

**Figure 7:**
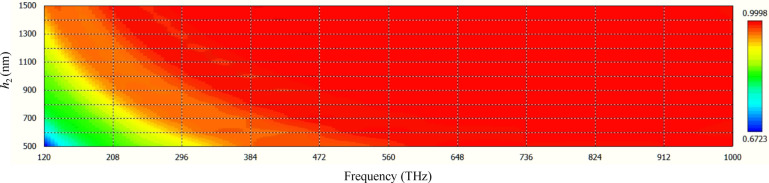
The absorption rate of the proposed solar cell for different cone heights *h*_2_.

The absorption rate of the proposed solar cell for different cone heights is investigated in Fig. 7. For covering the lower frequencies, the height of the cone should be increased. Since higher frequencies have more energy, the height can be shortened by partially covering the higher frequencies to reach a compact device. With the height of 500 nm (substrate thickness 100 nm), a 90% absorption rate for the frequencies beyond 300  THz can be achieved. The same performance is attained with metal-coated moth-eye films with the same geometry. The cone height and substrate thickness are respectively around 200 nm and 5 μm in this device^47^.

**Figure 8:**
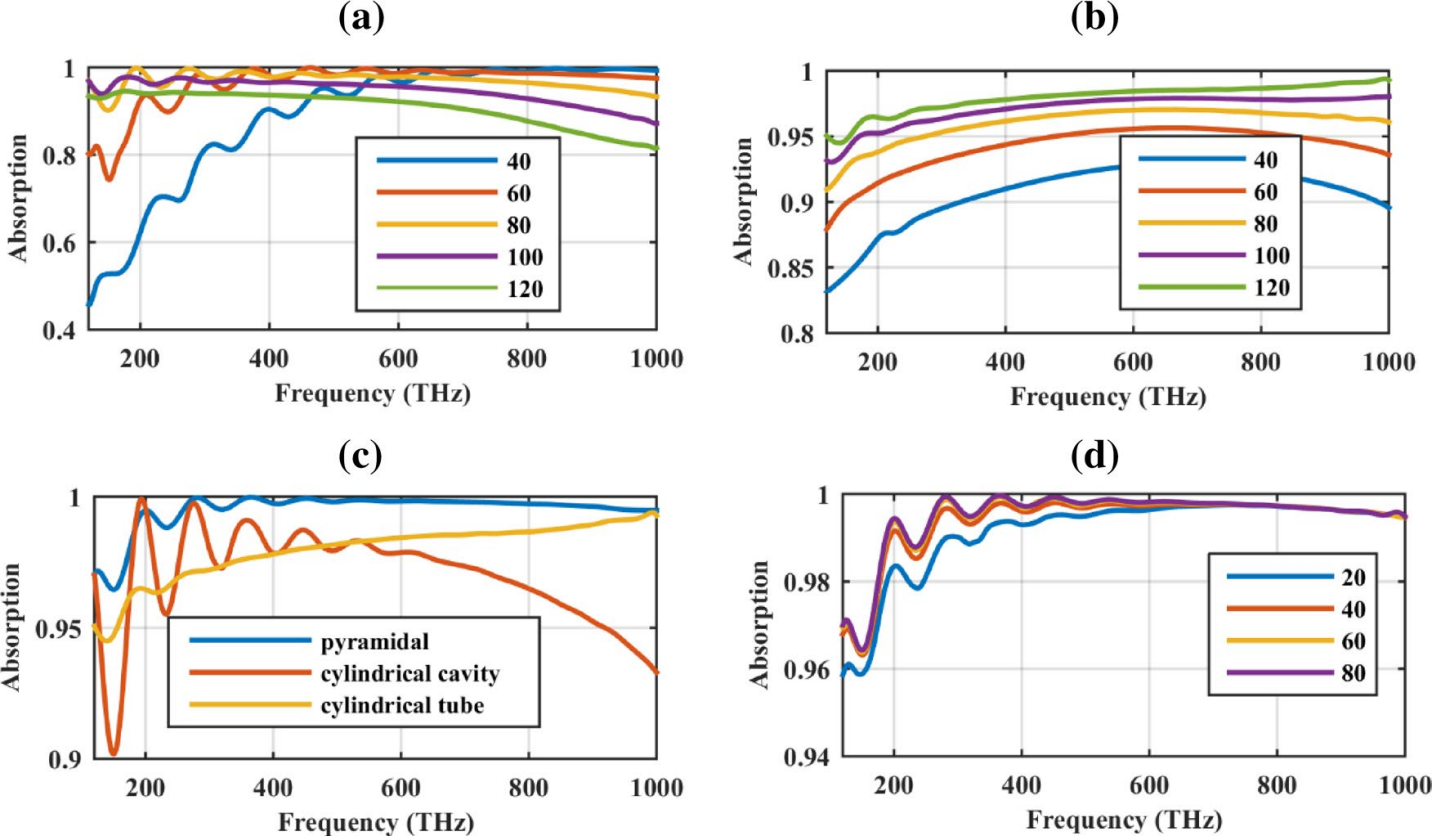
The absorption rate of the cylindrical (**a**) cavity and (**b**) tube for different thicknesses. (**c**) Comparison of the absorption rate of pyramidal solar cell with the optimum cylindrical cavity and tube solar cell. (**d**) The impact of the substrate thickness in the absorption rate of the pyramidal absorber.

Finally, the morphology-dependent behavior of the proposed structure is investigated in Fig. 8. Notably, tapering of individual nano-objects is regarded as a promising strategy to engineer the far-field and near-field optical response of nanostructured metamaterials^48–51^. However, since the nanowire system is relatively easy to realize and control, two simpler geometries are analyzed in the following. Specifically, in Fig. 8a the pyramidal nano-pillar is converted to a cylindrical cavity with the same height and shell thickness and the radius of the cavity is varied from 40 to 120 nm. The absorption rate is large at low (high) frequencies for large (small) radii, as expected. Thus, the absorption rate can be sacrificed to attain a simpler geometry. For the intermediate radius of 80 nm, the absorption rate above 90% in the solar spectrum is attained. Moreover, illustrated in Fig. 8b is the absorption rate of the same structure by converting it to a hollow tube. It is observed that by increasing the tube radius, the absorption increases as well, being above 95% in the solar spectrum for the radius of 120 nm. To further illustrate the priority of the pyramidal geometry, the absorption rate of the pyramidal geometry is compared with those of cylindrical cavity (with the radius 80 nm) and cylindrical tube (with the radius 120 nm) in Fig. 8c. Moreover, the substrate thickness of the pyramidal geometry is varied in Fig. 8d, to confirm that it is chosen properly. Increasing the substrate height beyond 40 nm has a negligible impact on the absorption rate.

## Conclusions

With the combined use of shell-shaped graphene-based hollow nano-pillars and a refractory metal substrate, a full-spectrum solar cell is designed. These two sections are respectively responsible for impedance matching and transmission blockage, leading to efficient energy absorption. Moreover, the performance is further explained by exhibiting the azimuthal and longitudinal cavity resonances with different orders in the spectrum. The polarization in-sensitive absorption of the solar cell is maintained up to 65°. Moreover, it is observed that tip truncation and using hollow particles are two key factors for the efficient absorption of the low-frequency waves. The proposed device is heat tolerant and environmentally robust and can be possibly realized by the current fabrication technology.

## Methods

The unit cell analysis of CST software is used for simulation^52^. The Floquet ports with two prorogating modes are considered during the simulations. The absorption rate (*A*) of the structure is calculated using the simulated reflectance (*R*) and transmittance (*T*) as^37^:

4 $$ A = 1 - T - R = 1 - \left| {S_{21} } \right|^{2} - \left| {S_{11} } \right|^{2} $$

where *S*_11_ and *S*_12_ are the scattering parameters.
